# Measuring patients’ experience of clinical trials: results of an exploratory review and stakeholder workshop

**DOI:** 10.1186/1745-6215-16-S2-P113

**Published:** 2015-11-16

**Authors:** Claire Planner

**Affiliations:** 1 University of Manchester, Manchester, UK

## Background

Measuring and responding to patient experience in clinical services is a policy priority, but this focus has not extended to the research setting. At the same time, with only 45% of trials recruiting to target, increasing recruitment and retention is a priority. It is hypothesised that using patient experience of trials data to improve the design of trials may have an impact on these outcomes. The study explored current measurement of patient experience of trials and stakeholder views around the feasibility of collecting and using this type of data.

## Methods

A scoping review of literature published 1995-2014 was conducted using databases, grey literature, and pharmaceutical reports. Information extracted from papers included a description of measures; how and when they were used; and aspects of patient experience measured. Expert stakeholder workshops were held in June and September 2014.

## Results

* Only 5 patient experience measures were identified (4 published; 1 unpublished); only 1 in a UK trial

* 3/5 measures were completed at trial completion; 2/5 included measurement ‘within trial’

* 4/5 were self-report; 1/5 was delivered face to face by a trialist

* Aspects measured included overall satisfaction; willingness to participate again; and willingness to recommend to friends and family

* Stakeholders agreed that implementing patient experience of trials measurement is feasible.

## Implications

Despite stakeholders recognising the potential use of patient experience data, few trials currently assess patient experience consistently. Research needs to establish how to measure and use trial experience data to improve experience, recruitment and retention.

